# Emerging PFAS contaminants PFNA and PFSA amplify epigenetic aging: sex- and age-stratified risks in an aging population

**DOI:** 10.3389/fragi.2025.1722675

**Published:** 2026-02-26

**Authors:** Ya-Qian Xu, Chongyu Ding, Hui Zhang, Yulu Gong, Darong Hao, Xuetong Zhao, Kai Li, Xiangwei Li

**Affiliations:** 1 School of Global Health, Chinese Centre for Tropical Diseases Research, Shanghai Jiao Tong University School of Medicine, Shanghai, China; 2 Hainan International Medical Center, Shanghai Jiao Tong University School of Medicine, Qionghai, Hainan, China; 3 School of Public Health, Shanghai Jiao Tong University School of Medicine, Shanghai, China; 4 Department of Ecological Environment, Yangtze Delta Region Institute of Tsinghua University, Jiaxing, Zhejiang, China

**Keywords:** PFAS, DNA methylation aging, GrimAge, sex disparities, NHANES

## Abstract

Emerging perfluoroalkyl substances (PFAS), such as perfluorononanoic acid (PFNA) and perfluorosulfonic acid (PFSA), are pervasive environmental contaminants that may influence human health. Their effects on aging, particularly through recently developed robust DNA methylation (DNAm) aging algorithms, remain largely unexplored. Using data from 326 U.S. adults aged ≥50 years (NHANES 1999–2000), we quantified serum PFAS via isotope-dilution tandem mass spectrometry and computed 12 DNAm aging algorithms. Weighted multivariable linear regression models assessed PFAS-DNAm aging associations, stratified by sex and age. PFNA exposure was significantly associated with DNAm algorithms via GrimAgeMortacc (β = 2.74, 95% CI: 1.04–4.44) and GrimAge2Mortacc (β = 2.45, 0.60–4.31), with stronger magnitudes in males (*P*-interaction = 0.0050) and adults aged 50–64 (*P*-interaction = 0.0270). PFSA was associated with LinAgeacc (β = 4.17, 0.60–7.74). Sex-stratified analyses revealed male-specific PFNA-HorvathAgeacc associations (β = 4.11, 0.71–7.52). These findings suggest that PFNA and PFSA may drive epigenetic aging disparities in aging populations, with males and middle-aged individuals at heightened risk. These findings underscore the need to regulate emerging PFAS and integrate epigenetic biomarkers into environmental health risk assessments.

## Graphical Abstract

**Figure:**
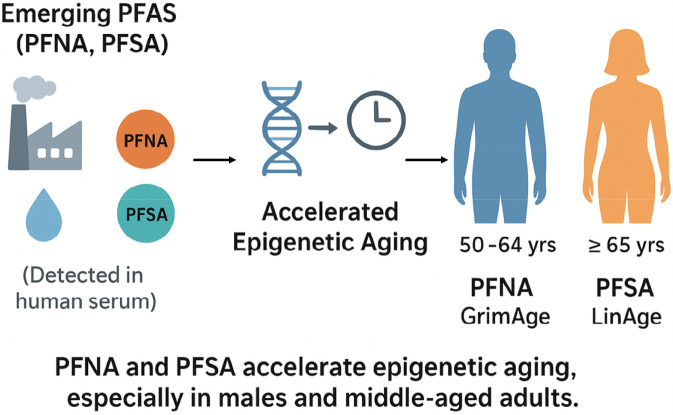

## 1 Introduction

Polyfluoroalkyl substances (PFAS) are a class of synthetic compounds renowned for their chemical stability and resistance to environmental degradation (Noori et al., 2025). These persistent chemicals have emerged as a significant public health concern due to their widespread contamination of drinking water supplies, affecting over 200 million U.S. residents (EWG, 2020). With biological persistence reflected in serum half-lives of up to 8.5 years, PFAS exposure has been linked to a range of adverse health outcomes (Rosato et al., 2024). Epidemiological evidence suggests associations between increases in exposure to specific PFAS and certain health impacts, such as increases in cholesterol levels (perfluorooctanoic acid (PFOA), Perfluorooctane sulfonic acid (PFOS), perfluorononanoic acid (PFNA), perfluorodecanoic acid (PFDA)) (Henn et al., 2024), lower antibody response to some vaccines (PFOA, PFOS, perfluorohexane sulfonic acid (PFHS), PFDA) (Vorst et al., 2021), changes in liver enzymes (PFOA, PFOS, PFHS) (Solan et al., 2023), pregnancy-induced hypertension and preeclampsia (PFOA, PFOS) (Preston et al., 2022), small decreases in birth weight (PFOA, PFOS) (Domingo, 2025), and increased risks of kidney and testicular cancers (PFOA) (Del Fiore et al., 2022).

Experimental studies have shown that PFAS can induce DNA methylation (DNAm) alterations in genes regulating oxidative stress and inflammation, which are pathways central to biological aging (Kim et al., 2021; Starling et al., 2020). Consistent with these mechanistic findings, epidemiological studies have further reported that each ln-unit increase in PFOA is associated with 20.41% (95% CI: −30.44%, −8.93%) shorter telomere length in spring-born infants, and that mothers in the mid and high PFOA tertiles exhibit 11.69% and 10.71% shorter telomere length in their spring-born infants, respectively. Advances in epigenetic profiling have revolutionized aging research by quantifying biological age through epigenetic “clocks.” These include first-generation epigenetic clocks (e.g., Horvath, Hannum) (Horvath, 2013; Hannum et al., 2013), second-generation models (e.g., PhenoAge, GrimAge, GrimAge2Mort) (Lu et al., 2022; Lu et al., 2019a; Levine et al., 2018), and emerging third-generation measures such as DunedinPACE (Belsky et al., 2020). The discrepancy between epigenetic age and chronological age, termed age acceleration (AA), has been widely used to assess the impact of certain factors on aging. AA has gained popularity in recent years for its ability to capture systemic aging processes more comprehensively than telomere length alone (Bejaoui et al., 2023). Despite some studies reporting on the impact of PFAS on telomere length, systematic investigations into the relationship between PFAS and various epigenetic “clocks” remain limited (Chaney and Wiley, 2023; Goodrich et al., 2021).

To address critical gaps in understanding the environmental determinants of biological aging, we leveraged the National Health and Nutrition Examination Survey (NHANES) 1999–2000—a nationally representative sample of U.S. older adults (age ≥50 years, n = 326) with measured serum PFAS concentrations and 12 DNAm aging algorithms (HorvathAgeAcc (Horvath, 2013), HannumAgeAcc (Hannum et al., 2013), SkinBloodAgeAcc (Hor et al., 2018), PhenoAgeAcc (Levine et al., 2018), LinAgeAcc (Lin et al., 2016), WeidnerAgeAcc (Weidner et al., 2014), VidalBraloAgeAcc (Vidal-Bralo et al., 2016), ZhangAgeAcc (Zhang et al., 2019), DunedinPoAm (Belsky et al., 2020), HorvathTelo (Lu et al., 2019b), GrimAgeMortAcc (Lu et al., 2019a), GrimAge2MortAcc (Lu et al., 2022)). Our study aims to (1) evaluate associations between six prevalent PFAS compounds (with detection frequencies >75%) and multi-dimensional epigenetic aging metrics and (2) assess age- and sex-stratified associations. This investigation seeks to advance our understanding of the environmental influences on biological aging and inform targeted interventions to protect vulnerable population subgroups.

## 2 Materials and methods

### 2.1 Study population

This study analyzed data from the NHANES, a comprehensive program conducted by the National Center for Health Statistics (NCHS) to assess the health and nutritional status of the U.S. civilian noninstitutionalized population (Bryan et al., 2021). NHANES employs a complex, multi-stage probability sampling design to ensure a representative sample of the civilian, non-institutionalized U.S. population. Each cycle recruits distinct cohorts through a stratified, multistage sampling process, with oversampling of certain demographic groups, including older adults (≥50 years), racial/ethnic minorities (e.g., non-Hispanic Black, Mexican American), and low-income populations, to enhance statistical power for subgroup analyses.

For this analysis, data from the 1999–2000 cycle were used. Among the 9,965 participants in this cycle, 8,745 were excluded due to the lack of DNAm data, which was only available for adults aged 50 and older. An additional 894 were excluded due to missing information on PFAS concentrations. Ultimately, 326 participants aged ≥50 years were included in the analysis.

### 2.2 Ethics approval and consent to participate

All participants provided written informed consent, and the study protocol was approved by the National Center for Health Statistics (NCHS) Research Ethics Review Board. As this analysis used de-identified data with no direct participant interaction, it was classified as exempt from institutional review board review, in accordance with National Institutes of Health policy.

### 2.3 Quantification of serum PFAS concentrations

Serum concentrations of eleven PFASs were measured during 1999–2000 by NHANES at the CDC’s Division of Laboratory Sciences (NHANES, 2022). A multiple reaction monitoring (MRM) experiment was used to measure the following analytes: perfluorooctane sulfonamide (PFSA), 2-(N-ethyl-perfluorooctane sulfonamido) acetic acid (EPAH), 2-(N-methyl-perfluorooctane sulfonamido) acetic acid (MPAH), perfluorohexane sulfonic acid (PFHS), PFOS, PFOA, Perfluoroheptanoic acid (PFHP), PFNA, perfluorodecanoic acid (PFDE), perfluoroundecanoic acid (PFUA), and perfluorododecanoic acid (PFDO). For clarity, these original NHANES abbreviations correspond to the currently accepted standard nomenclature as follows: PFSA = PFOSA, EPAH = Et-PFOSA-AcOH, and MPAH = Me-PFOSA-AcOH.

In summary, serum samples were diluted with 0.1 M formic acid, and analytes were concentrated on a C_18_ solid-phase extraction column before chromatographic separation on a C_8_ high-performance liquid chromatography (HPLC) column. Detection was performed using negative-ion TurboIonSpray ionization and tandem mass spectrometry. Isotope-labeled internal standards (^18^O_2_-PFSA, ^18^O_2_-PFOS, and ^13^C_2_-PFOA) were used for quantification. Calibration standards were spiked into calf serum to account for matrix effects. The method’s limit of detection (Tang et al., 2017) was calculated using 3S0, with accuracies ranging from 77% to 109%. Sensitivity was verified through calibration curves and replicate analyses, with coefficients of variation maintained below 6%–16%.

Only targets with detection frequencies (DF) above 75% (EPAH, MPAH, PFHS, PFNA, PFOA, PFOS, PFSA) were included in the statistical analysis.

### 2.4 Assessments of DNA methylation aging algorithms

As outlined in the NHANES DNA Methylation Array and Epigenetic Biomarkers Data Documentation (NHANES, 2024), DNAm profiles were generated from whole blood samples using the Illumina Infinium HumanMethylationEPIC BeadChip (EPIC array) for a subset of adults aged 50 years and older. This subset included approximately half of the eligible non-Hispanic White participants, along with all eligible non-Hispanic Black, Mexican American, other Hispanic, and participants of other races. Measurements were performed in Dr. Yongmei Liu’s laboratory at Duke University, following the manufacturer’s instructions. In brief, raw data preprocessing and normalization were conducted using the minfi package in R, incorporating background correction, dye bias adjustment, and beta-value calculation.

Twelve DNAm algorithms were calculated based on the aforementioned DNAm profiles and provided by the NHANES dataset. To ensure comprehensive coverage of epigenetic aging measures, we selected all 12 DNAm algorithms included in the NHANES dataset, which are:

1. HorvathAge (pan-tissue clock) (Horvath, 2013),
2. HannumAge (leukocyte-specific clock) (Hannum et al., 2013),
3. SkinBloodAge (skin and blood-adjusted clock) (Hor et al., 2018),
4. PhenoAge (clinical biomarker-integrated clock) (Levine et al., 2018),
5. LinAge (lifespan prediction model) (Lin et al., 2016),
6. WeidnerAge (mitotic age estimator) (Weidner et al., 2014),
7. VidalBraloAge (immune cell-adjusted metric) (Vidal-Bralo et al., 2016),
8. ZhangAge (plasma protein composite clock) (Zhang et al., 2019),
9. DunedinPoAm (pace of aging biomarker) (Belsky et al., 2020),
10. HorvathTelo (telomere length proxy) (Lu et al., 2019b),
11. GrimAgeMort (mortality risk predictor) (Lu et al., 2019a),
12. GrimAge2Mort (updated GrimAge algorithm) (Lu et al., 2022).

To further refine the analysis, we computed age-adjusted versions of these 12 DNAm algorithms. This was achieved by subtracting each participant’s chronological age from their predicted biological age. The resulting metrics were labeled as HorvathAgeacc, HannumAgeacc, and similarly for the other biomarkers.

### 2.5 Covariates

Covariates were selected based on their potential confounding influence on PFAS exposure and epigenetic aging. As described previously (Muntner et al., 2020; Saint-Maurice et al., 2020), demographic variables included chronological age (continuous, years) and sex (male/female) was collected through a self-report questionnaire. Race and ethnicity were recorded using predefined categories to characterize the population and facilitate the oversampling of non-Hispanic Black and Mexican American individuals. Lifestyle factors encompassed smoking pack-years, estimated using a DNAm-based method (the Maas 13-CpGs model (Maas et al., 2019)), and alcohol consumption categorized as Never, Ever, Current, or Don’t know based on self-reported intake of ≥12 alcoholic beverages per year. Height and weight were objectively measured by trained staff following standardized protocols. Biological mediators included leukocyte composition, which was determined using the Houseman method based on DNAm data (Houseman et al., 2012). Biological mediators included leukocyte composition, which was determined using the Houseman method based on DNAm data. Serum C-reactive protein (CRP, log-transformed, mg/dL) was measured in blood samples using a latex-enhanced nephelometry method to account for systemic inflammation.

### 2.6 Statistical analysis

All analyses incorporated NHANES sampling weights to account for complex survey design and non-response bias. Demographic characteristics of the study participants were summarized by subset using standard descriptive methods. To address the skewed distribution of PFAS concentrations, we applied log-transformation to approximate normality. The geometric mean (GM) was used to summarize the central tendency of PFAS levels. Comparisons of geometric mean PFAS concentrations across sex and age groups were conducted using weighted t-tests on log-transformed values.

Correlations among the 12 DNAm algorithms, chronological age, and PFAS concentrations were assessed using Spearman correlation coefficients and were illustrated in a correlation matrix plot. Associations of PFAS with these 12 DNAm algorithms were assessed using weighted multivariable linear regression. Two hierarchical models were constructed: Model 1 adjusted for age, sex, and leukocyte composition. Model 2 further adjusted for race/ethnicity, educational level, poverty income ratio, and body mass index (BMI), and CRP.

Given the influence of sex and age on biological and epigenetic aging processes (Horvath and Raj, 2018; Unnikrishnan et al., 2019), stratified analyses evaluated effect modification by sex and age group (50–64 vs. ≥65 years) through interaction terms (PFAS × sex or PFAS × age group).

Statistical significance was defined as a two-tailed *P*-value < 0.05. All analyses were performed using SAS 9.4 (SAS Institute, Inc., Cary, NC) and R version 4.3.1, with visualization conducted in ggplot2.

## 3 Results

### 3.1 Study population

Table 1 provides an overview of the study population. Of the 326 participants included, the gender ratio was nearly balanced (51.53% males), and the average age (standard deviation, SD) was 66.87 (9.69) years. Approximately one-third of the participants were Non-Hispanic White, about 60% had less than 9 years of education, and about 60% were current drinkers. The means of BMI, smoking pack-years, and CRP levels were 28.94 kg/m^2^, 20.47 pack-years, and 0.74 mg/dL, respectively.

**TABLE 1:** Characteristics of study population.

Characteristics	NHANES 1999–2000
Age (years, mean ± SD)	66.87 ± 9.69
50–64 years	139^a^ (57.62 ± 4.53)
≥65 years	187 (73.75 ± 6.13)
Sex (N/%)	
Men	168 (51.53)
Women	158 (48.47)
Race/ethnicity, N (%)	
Non-Hispanic white	107 (32.82)
Non-Hispanic black	62 (19.02)
Mexican American	126 (38.65)
Other races-including multi-racial	7 (2.15)
Other hispanic	24 (7.36)
Educational levels (N/%)^b^	
Low (≤9 years)	188 (57.67)
Intermediate (10–11 years)	57 (17.48)
High (≥12 years)	80 (24.54)
BMI (kg/m^2^, mean ± SD)^c^	28.94 ± 5.56
Smoking pack years (pack-year, mean ± SD)	20.47 ± 13.40
Alcohol consumption (N/%)^a^	
Never	60 (18.40)
Ever	58 (17.79)
Current	186 (57.06)
Don’t know	22 (6.75)
C-reactive protein (mg/dL)	0.74 ± 1.98

Abbreviations: SD, standard deviation.

^a^ The first number in each age group column represents the number of participants in that subgroup.

^b^ Data missing for 1 participant.

^c^ DNA, methylation predicted pack years of smoking.

### 3.2 Serum PFAS profiles

Supplementary Table S1 summarizes the distribution of PFASs in the serum of the study participants. EPAH, MPAH, PFHS, PFNA, PFOA, PFOS, and PFSA were detected in high frequencies across all participants, with DFs of 84.7%, 94.2%, 99.7%, 95.4%, 100%, 100%, and 95.7%, respectively. In contrast, PFUA, PFDE, PFDO, and PFHP had DFs of less than 40%. The GM values for these PFASs were as follows: EPAH (0.53 ng/mL), MPAH (0.62 ng/mL), PFHS (1.84 ng/mL), PFNA (0.55 ng/mL), PFOA (4.08 ng/mL), PFOS (28.72 ng/mL), and PFSA (0.29 ng/mL). There were no significant differences between sexes (male vs. female) or age groups (50–64 vs. ≥ 65) for these compounds (Supplementary Tables S2 and S3).

### 3.3 Profiles of DNAm algorithms

Table 2 presents the 12 DNAm aging algorithms for all participants, categorized by sex and age. The acceleration values for these DNAm algorithms ranged from −12.88 (WeidnerAgeacc) to 6.54 (HorvathTelo). Significant differences were observed (*P*-values: <0.0001–0.0261). Specifically, HorvathAgeacc, DunedinPoAm, GrimAgeMortacc, and GrimAge2Mortacc exhibited higher accelerations in males compared to females, while the opposite trend was observed for HorvathTelo. Among participants aged 50–64 years, greater accelerations were observed in comparison to those aged ≥65 years for several algorithms, including HorvathAgeacc, HannumAgeacc, SkinBloodAgeacc, PhenoAgeacc, WeidnerAgeacc, VidalBraloAgeacc, ZhangAgeacc, HorvathTelo, GrimAgeMortacc, and GrimAge2Mortacc, with *P*-values ranging from <0.0001 to 0.0002.

**TABLE 2:** Twelve DNAm aging algorithms for all participants, categorized by sex and age.

DNAm aging algorithm	Total (N = 326)	By sex			By age		
		Males (n = 168)	Females (n = 158)	*P*-value	Aged 50–64 years (n = 139)	Aged ≥65 years (n = 187)	*P*-value
HorvathAgeacc	−0.01 ± 6.77	0.80 ± 6.88	−0.86 ± 6.56	0.0261	2.93 ± 4.96	−2.19 ± 7.11	<0.0001
HannumAgeacc	1.00 ± 6.92	1.72 ± 6.89	0.23 ± 6.88	0.0513	3.41 ± 5.40	−0.79 ± 7.38	<0.0001
SkinBloodAgeacc	−1.90 ± 6.65	−1.71 ± 6.83	−2.11 ± 6.48	0.5898	−0.19 ± 4.25	−3.18 ± 7.75	<0.0001
PhenoAgeacc	−10.46 ± 7.89	−10.51 ± 8.03	−10.41 ± 7.77	0.903	−8.58 ± 6.36	−11.86 ± 8.61	0.0002
LinAgeacc	−9.66 ± 8.60	−9.22 ± 8.56	−10.11 ± 8.65	0.3515	−8.67 ± 7.28	−10.39 ± 9.42	0.0745
WeidnerAgeacc	−12.88 ± 10.16	−13.09 ± 10.18	−12.65 ± 10.16	0.6992	−8.66 ± 7.05	−16.01 ± 10.97	<0.0001
VidalBraloAgeacc	−6.52 ± 8.03	−5.74 ± 8.28	−7.35 ± 7.68	0.0695	−0.85 ± 5.38	−10.74 ± 7.01	<0.0001
ZhangAgeacc	−0.01 ± 7.01	0.15 ± 6.89	−0.19 ± 7.16	0.6636	6.29 ± 3.14	−4.70 ± 5.18	<0.0001
DunedinPoAm	1.12 ± 0.09	1.14 ± 0.09	1.11 ± 0.10	0.0085	1.13 ± 0.10	1.12 ± 0.09	0.5925
HorvathTelo	6.54 ± 0.32	6.48 ± 0.32	6.60 ± 0.31	0.0005	6.69 ± 0.26	6.43 ± 0.31	<0.0001
GrimAgeMortacc	0.39 ± 5.68	2.28 ± 5.69	−1.63 ± 4.93	<0.0001	2.99 ± 5.37	−1.54 ± 5.11	<0.0001
GrimAge2Mortacc	6.30 ± 6.20	7.81 ± 6.34	4.70 ± 5.64	<0.0001	9.26 ± 5.75	4.10 ± 5.59	<0.0001

### 3.4 Correlations of DNAm algorithms, chronological age, and PFAS concentrations

In the overall population (Supplementary Figure S1), only PFOA showed a significant correlation with chronological age (r = −0.13, *P* = 0.0203). PFOA had significant correlations with VidalBraloAgeacc (r = 0.13, *P* = 0.0233) and ZhangAgeacc (*P* = 0.0290, r = 0.12). PFSA exhibited a significant correlation with GrimAge2Mortacc (r = −0.12, *P* = 0.0327).

**FIGURE 1:**
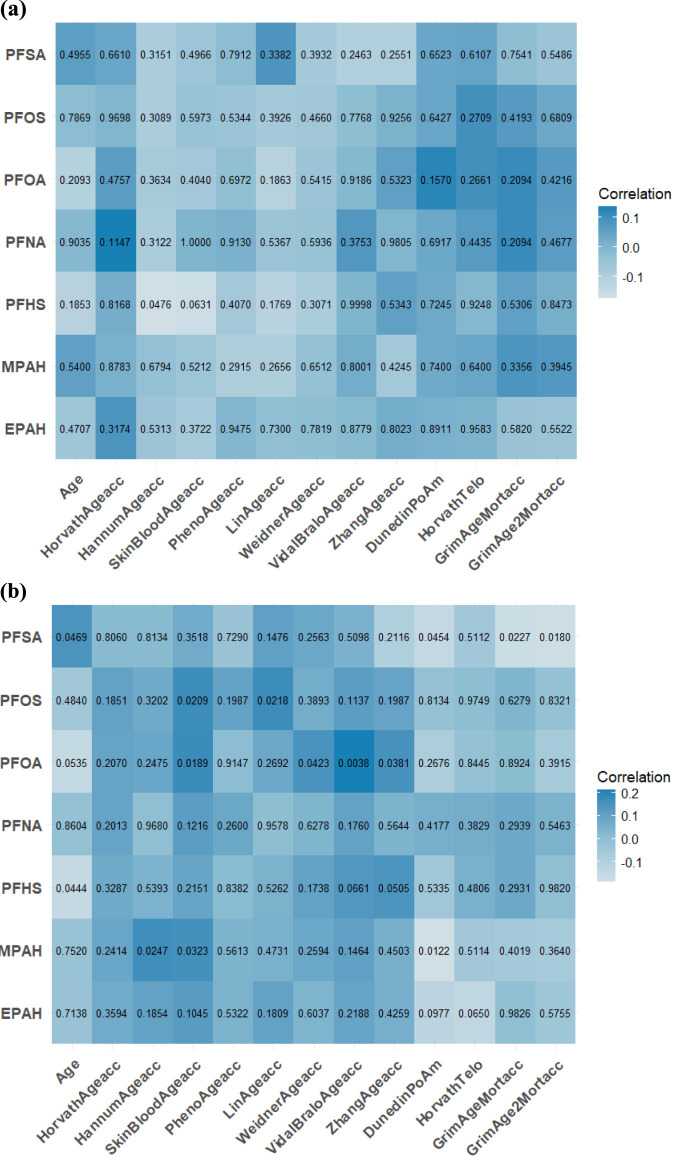
**(a)** Spearman correlation coefficients of age, polyfluoroalkyl chemicals and 12 DNAm aging algorithms at NHANES 1999–2000 in 50–64 years; **(b)** Spearman correlation coefficients of age, polyfluoroalkyl chemicals and 12 DNAm aging algorithms at NHANES 1999–2000 in ≥65 years.

**FIGURE 2:**
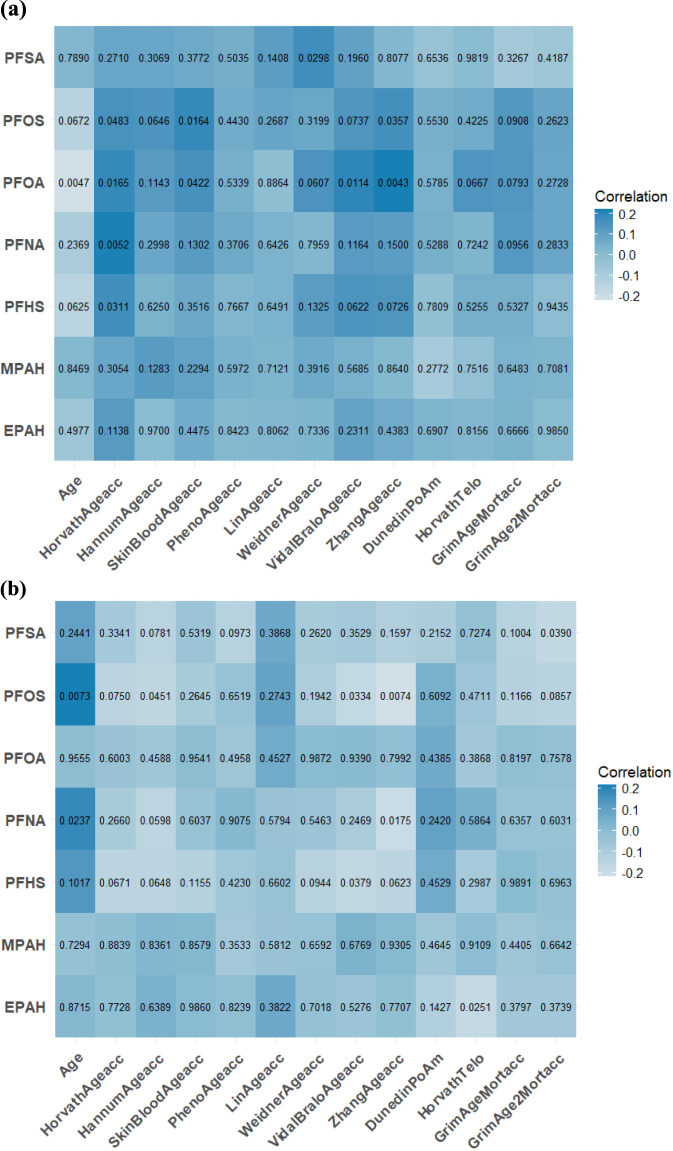
**(a)** Spearman correlation coefficients of age, polyfluoroalkyl chemicals and 12 DNAm aging algorithms at NHANES 1999–2000 in male; **(b)** Spearman correlation coefficients of age, polyfluoroalkyl chemicals and 12 DNAm aging algorithms at NHANES 1999–2000 in female.

Figure 1 displays the Spearman correlation coefficients for 12 DNAm algorithms chronological age, and PFASs, and the among participants aged 50–64 (Figure 1a) and ≥65 years (Figure 1b). In the 50–64 age group, PFHS was significantly correlated with HannumAgeacc (r = −0.17, *P* = 0.0476). In the ≥65 group, PFSA correlated with DunedinPoAm (r = −0.15, *P* = 0.0454), GrimAgeMortacc (r = −0.17, *P* = 0.0227), and GrimAge2Mortacc (r = −0.18, *P* = 0.0180). Additionally, PFOA showed correlations with VidalBraloAgeacc (r = 0.21, *P* = 0.0038). Sex-stratified analyses (Figure 2) revealed distinct patterns. In males, PFOA significantly correlated with HorvathAgeacc (r = 0.19, *P* = 0.0165), SkinBloodAgeacc (r = 0.16, *P* = 0.0422), VidalBraloAgeacc (r = 0.20, *P* = 0.0114), and ZhangAgeacc (r = 0.22, *P* = 0.0043). PFOS correlated with HorvathAgeacc (r = 0.16, *P* = 0.0483), SkinBloodAgeacc (r = 0.19, *P* = 0.0164) and ZhangAgeacc (r = 0.17, *P* = 0.0357). PFSA correlated with WeidnerAgeacc (r = 0.17, *P* = 0.0298). In females, PFNA correlated with ZhangAgeacc (r = −0.19, *P* = 0.0175). PFOS correlated with HannumAgeacc (r = −0.16, *P* = 0.0451), VidalBraloAgeacc (r = −0.17, *P* = 0.0334), and ZhangAgeacc (r = −0.22, *P* = 0.0074). PFSA correlated with GrimAge2Mortacc (r = −0.17, *P* = 0.0390).

### 3.5 Associations of PFASs with DNAm algorithms

Table 3 displays the associations between PFNA and PFSA with the 12 DNAm algorithms. After multivariable adjustment, PFNA exhibited significant associations with DunedinPoAm, GrimAgeMortacc, and GrimAge2Mortacc, with *P*-values of 0.0157, 0.0017, and 0.0100, and β coefficients of 0.04 (0.01, 0.07), 2.74 (1.04, 4.44), and 2.45 (0.60, 4.31), respectively. PFSA showed a significant association only with LinAgeacc (*P* = 0.0230, β = 4.17, 95% CI: 0.60–7.74). No significant associations were observed for EPAH, MPAH, PFHS, PFOA, or PFOS with any DNAm algorithm (Supplementary Table S4).

**TABLE 3:** Associations of PFNA and PFSA with 12 DNA methylation algorithms of aging.

DNAm aging algorithm	Model 1		Model 2	
	β (95% CI)	*P*-value	β (95% CI)	*P*-value
PFNA				
HorvathAgeacc	1.79 (−0.38, 3.96)	0.1076	1.92 (−0.30, 4.14)	0.0911
HannumAgeacc	0.48 (−1.73, 2.69)	0.6729	0.81 (−1.43, 3.06)	0.4783
SkinBloodAgeacc	1.66 (−0.62, 3.93)	0.1537	1.68 (−0.58, 3.94)	0.1460
PhenoAgeacc	1.62 (−0.97, 4.21)	0.2210	1.84 (−0.84, 4.52)	0.1793
LinAgeacc	0.90 (−2.08, 3.87)	0.5545	0.93 (−2.16, 4.01)	0.5571
WeidnerAgeacc	1.09 (−2.18, 4.37)	0.5134	0.77 (−2.69, 4.23)	0.6625
VidalBraloAgeacc	1.61 (−0.31, 3.53)	0.1021	1.47 (−0.60, 3.53)	0.1647
ZhangAgeacc	0.40 (−0.45, 1.25)	0.3586	0.37 (−0.45, 1.19)	0.3782
DunedinPoAm	0.03 (−0.01, 0.06)	0.0979	0.04 (0.01, 0.07)	0.0157
HorvathTelo	0.03 (−0.06, 0.13)	0.4555	0.02 (−0.08, 0.11)	0.7582
GrimAgeMortacc	2.31 (0.74, 3.88)	0.0042	2.74 (1.04, 4.44)	0.0017
GrimAge2Mortacc	1.88 (0.14, 3.62)	0.0353	2.45 (0.60, 4.31)	0.0100
PFSA				
HorvathAgeacc	0.53 (−2.01, 3.06)	0.6850	0.89 (−1.71, 3.49)	0.5024
HannumAgeacc	−0.15 (−2.72, 2.43)	0.9111	0.65 (−1.97, 3.28)	0.6259
SkinBloodAgeacc	0.28 (−2.38, 2.94)	0.8370	0.84 (−1.81, 3.48)	0.5372
PhenoAgeacc	−0.94 (−3.96, 2.08)	0.5424	−0.25 (−3.38, 2.89)	0.8784
LinAgeacc	3.33 (−0.12, 6.77)	0.0593	4.17 (0.60, 7.74)	0.0230
WeidnerAgeacc	2.34 (−1.47, 6.15)	0.2288	1.51 (−2.52, 5.54)	0.4638
VidalBraloAgeacc	1.63 (−0.61, 3.87)	0.1554	1.30 (−1.11, 3.71)	0.2920
ZhangAgeacc	0.13 (−0.87, 1.13)	0.7985	0.52 (−0.44, 1.48)	0.2906
DunedinPoAm	−0.02 (−0.06, 0.01)	0.2128	−0.01 (−0.05, 0.03)	0.6097
HorvathTelo	0.02 (−0.09, 0.13)	0.7338	0.02 (−0.10, 0.13)	0.7966
GrimAgeMortacc	−0.73 (−2.57, 1.12)	0.4425	−0.82 (−2.84, 1.20)	0.4253
GrimAge2Mortacc	−1.32 (−3.36, 0.71)	0.2038	−1.25 (−3.43, 0.94)	0.2650

Model 1: adjusted for age, sex, white blood cell count.

Model 2: adjusted for age, sex, white blood cell count, race/ethnicity, educational status, poverty income ratio, and body mass index, C-reactive protein.

Age-stratified analyses (Table 4) revealed significant associations between PFNA and GrimAgeMortacc (*P* = 0.0140) and GrimAge2Mortacc (*P* = 0.0174) in participants aged 50–64, with β values of 3.93 (0.85, 7.01) for GrimAgeMortacc and 4.00 (0.76, 7.25) for GrimAge2Mortacc. In the ≥65 age group, PFNA was also significantly associated with GrimAgeMortacc (β = 2.60, 95% CI: 0.54–4.66, *P* = 0.0144), while PFSA was significantly associated with LinAgeacc (β = 6.24, 95% CI: 0.44–12.04, *P* = 0.0366). EPAH, MPAH, PFHS, PFOA, and PFOS showed no significant associations in the age-stratified analyses (Supplementary Table S5).

**TABLE 4:** Associations of polyfluoroalkyl chemicals with 12 DNA methylation algorithms of aging by age.

DNAm aging algorithm	50–64 years		≥65 years		*P*-^interaction^
	β (95% CI)	*P*-value	β (95% CI)	*P*-value	
PFNA					
HorvathAgeacc	1.42 (−2.02, 4.87)	0.4197	2.30 (−0.77, 5.37)	0.1434	0.1128
HannumAgeacc	−1.10 (−4.52, 2.33)	0.5314	1.96 (−1.11, 5.03)	0.2134	0.3040
SkinBloodAgeacc	−0.15 (−3.05, 2.74)	0.9173	2.97 (−0.33, 6.28)	0.0797	0.0977
PhenoAgeacc	0.14 (−3.73, 4.01)	0.9431	3.02 (−0.74, 6.78)	0.1180	0.1078
LinAgeacc	−1.64 (−6.31, 3.02)	0.4908	2.38 (−1.86, 6.62)	0.2727	0.4001
WeidnerAgeacc	−0.56 (−5.38, 4.26)	0.8206	2.57 (−2.34, 7.47)	0.3070	0.6580
VidalBraloAgeacc	0.37 (−2.57, 3.31)	0.8040	2.42 (−0.49, 5.33)	0.1055	0.1948
ZhangAgeacc	−0.45 (−1.44, 0.54)	0.3716	0.78 (−0.43, 2.00)	0.2082	0.2326
DunedinPoAm	0.06 (−0.01, 0.12)	0.0755	0.03 (−0.01, 0.07)	0.1459	0.0551
HorvathTelo	0.02 (−0.13, 0.18)	0.7676	0.00 (−0.13, 0.13)	0.9710	0.9161
GrimAgeMortacc	3.93 (0.85, 7.01)	0.0140	2.60 (0.54, 4.66)	0.0144	0.0057
GrimAge2Mortacc	4.00 (0.76, 7.25)	0.0174	2.15 (−0.13, 4.43)	0.0665	0.0270
PFSA					
HorvathAgeacc	−0.33 (−3.53, 2.86)	0.8378	1.90 (−2.37, 6.17)	0.3841	0.4523
HannumAgeacc	−1.43 (−4.60, 1.74)	0.3780	2.27 (−1.99, 6.53)	0.2976	0.3699
SkinBloodAgeacc	−1.48 (−4.15, 1.19)	0.2789	3.00 (−1.59, 7.60)	0.2024	0.2942
PhenoAgeacc	−2.07 (−5.63, 1.48)	0.2560	1.49 (−3.76, 6.73)	0.5790	0.8107
LinAgeacc	1.84 (−2.47, 6.15)	0.4049	6.24 (0.44, 12.04)	0.0366	0.0179
WeidnerAgeacc	−1.99 (−6.44, 2.45)	0.3818	6.09 (−0.66, 12.84)	0.0789	0.2605
VidalBraloAgeacc	−1.00 (−3.72, 1.71)	0.4716	3.45 (−0.58, 7.48)	0.0958	0.2258
ZhangAgeacc	−0.45 (−1.37, 0.47)	0.3364	1.35 (−0.33, 3.02)	0.1171	0.1211
DunedinPoAm	0.00 (−0.06, 0.05)	0.8998	−0.04 (−0.09, 0.02)	0.1648	0.4081
HorvathTelo	0.08 (−0.06, 0.23)	0.2750	−0.02 (−0.19, 0.16)	0.8676	0.9758
GrimAgeMortacc	−0.40 (−3.34, 2.54)	0.7902	−1.57 (−4.47, 1.33)	0.2901	0.4264
GrimAge2Mortacc	−0.93 (−4.01, 2.15)	0.5559	−1.98 (−5.16, 1.19)	0.2224	0.2838

Liner regressions were adjusted for sex, white blood cell count, race/ethnicity, educational status, poverty income ratio, and body mass index, C-reactive protein.

Sex-stratified analyses (Table 5) indicated that PFNA was significantly associated with HorvathAgeacc (β = 4.11, 95% CI: 0.71–7.52, *P* = 0.0195), GrimAgeMortacc (β = 3.69, 95% CI: 1.09–6.29, *P* = 0.0062), and GrimAge2Mortacc (β = 2.98, 95% CI: 0.24–5.72, *P* = 0.0346) among males, whereas no significant associations were observed in females. EPAH, MPAH, PFHS, PFOA, PFOS, and PFSA did not show significant associations with any DNAm algorithm in the sex-stratified analyses (Supplementary Table S6). In the overall sample, significant associations between PFNA and GrimAgeMortacc were identified (*P*-interaction = 0.0057), with a stronger association observed in males (*P*-interaction = 0.0050). Additionally, PFNA was significantly associated with GrimAge2Mortacc among participants aged 50–64 years (*P*-interaction = 0.0270) and in males (*P*-interaction = 0.0152).

**TABLE 5:** Associations of polyfluoroalkyl chemicals with 12 DNA methylation algorithms of aging by sex.

DNAm aging algorithm	Male		Female		*P*-^interaction^
	β (95% CI)	*P*-value	β (95% CI)	*P*-value	
PFNA					
HorvathAgeacc	4.11 (0.71, 7.52)	0.0195	0.12 (−2.61, 2.86)	0.9301	0.3098
HannumAgeacc	2.38 (−1.14, 5.89)	0.1871	−0.77 (−3.64, 2.11)	0.6024	0.7415
SkinBloodAgeacc	2.43 (−1.39, 6.25)	0.2142	0.79 (−1.79, 3.36)	0.5513	0.2417
PhenoAgeacc	1.45 (−2.57, 5.47)	0.4810	1.44 (−2.10, 4.99)	0.4268	0.1904
LinAgeacc	0.96 (−3.84, 5.76)	0.6961	1.19 (−2.69, 5.07)	0.5490	0.5399
WeidnerAgeacc	−0.31 (−5.38, 4.76)	0.9050	1.99 (−2.68, 6.67)	0.4051	0.3942
VidalBraloAgeacc	1.63 (−1.61, 4.87)	0.3254	1.23 (−1.40, 3.87)	0.3605	0.1488
ZhangAgeacc	0.78 (−0.63, 2.20)	0.2795	−0.12 (−1.00, 0.75)	0.7852	0.5761
DunedinPoAm	0.04 (−0.01, 0.09)	0.1169	0.04 (0.00, 0.09)	0.0596	0.0147
HorvathTelo	−0.04 (−0.19, 0.11)	0.5679	0.09 (−0.04, 0.22)	0.1816	0.5717
GrimAgeMortacc	3.69 (1.09, 6.29)	0.0062	1.89 (−0.38, 4.16)	0.1055	0.0050
GrimAge2Mortacc	2.98 (0.24, 5.72)	0.0346	1.94 (−0.63, 4.52)	0.1423	0.0152

Liner regressions were adjusted for age, white blood cell count, race/ethnicity, educational status, poverty income ratio, and body mass index, C-reactive protein.

## 4 Discussion

In this study, we evaluated and compared the associations of PFAS with 12 widely used aging DNAm algorithms in 326 U.S. adults. Our findings reveals that PFNA and PFSA accelerate epigenetic aging in a sex- and age-dependent manner. Specifically, PFNA exposure was robustly associated with GrimAgeacc, a DNAm biomarker predictive of mortality, particularly in males (β = 3.69, 95% CI: 1.09–6.29) and middle-aged individuals (β = 3.93, 0.85–7.01). PFSA, though less studied, exhibited distinct links to lipid metabolism-associated aging (LinAgeacc, β = 4.17, 0.60–7.74). These results highlight the compound-specific epigenetic toxicity of PFAS and suggest that regulatory focus should extend beyond widely studied legacy PFAS such as PFOA and PFOS to include additional long-chain PFAS like PFNA and PFSA, which are persistent, bioaccumulative, and have demonstrated toxic effects.

Our age-stratified results suggest midlife (50–64 years) as a critical window for PFNA-driven aging, which aligns with emerging evidence on the significant impact of environmental exposures during this period. Midlife is characterized by the onset of multiple age-related conditions including cardiovascular diseases, metabolic disorders, and early tissue degeneration (El Khoudary et al., 2020), making it a sensitive stage when environmental toxicants may disproportionately influence biological aging. This pattern may reflect the particular responsiveness of GrimAge-related DNAm sites to PFNA exposure, especially those regulating inflammation, DNA repair, cellular senescence, and protein metabolism. Importantly, PFNA has been shown to affect lipid transport, peroxisomal signaling, and oxidative stress pathways, which overlap with several DNAm modules embedded in GrimAge and GrimAge2Mortacc. These clocks incorporate CpG sites associated with inflammatory cytokines, smoking-related damage, and mortality-linked plasma proteins, which may help explain their heightened sensitivity to PFNA-induced epigenetic alterations. The lack of PFOS/PFOA associations in this study contrasts with their established links to cardiovascular disease (Schlezinger and Gokce, 2024), possibly due to GrimAge is based on a set of DNAm sites that may be more sensitive to the types of environmental exposures associated with PFNA.

The immunotoxicity of PFAS, including PFNA and PFSA, has been linked to various pathways. For instance, PFAS can modulate osteoimmunology via Peroxisome Proliferator-Activated Receptor Gamma (PPARγ), potentially influencing immune responses through osteoclast and osteoblast imbalances (Cao and Ng, 2025). Additionally, experimental studies have shown that PFAS can disrupt calcium signaling in immune cells, leading to increased inflammation and tissue injury (He et al., 2025). These mechanisms may underpin the observed associations between PFAS exposure and accelerated epigenetic aging (Zhao Z. et al., 2024). Furthermore, PFAS exposure has been associated with a wide range of health effects, including increased cholesterol levels, lower antibody response to vaccines, changes in liver enzymes, pregnancy-induced hypertension, and certain cancers (Henn et al., 2024; Gruber, 2025; Liu et al., 2025; Dangudubiyyam et al., 2024). These health effects may be mediated through epigenetic modifications, particularly DNAm, which can influence gene expression and cellular function (Sutter et al., 2024; Lawson et al., 2025).

Beyond these mechanisms, mounting evidence suggests that the mTOR signaling pathway may represent an additional mechanistic link between PFNA/PFSA exposure and alterations in DNAm aging clocks. mTOR is a central regulator of cellular growth, metabolism, autophagy, and organismal aging. Nuclear mTOR signaling directly shapes transcriptional programs involved in metabolic homeostasis (Zhao T. et al., 2024), while amino acid–responsive mTOR activation influences glucose regulation and downstream chromatin remodeling (Fan et al., 2022). Dysregulated mTOR signaling has been implicated in age-related muscle dysfunction, impaired protein synthesis, and systemic metabolic decline (Fan et al., 2025). PFAS have been shown to interfere with nutrient-sensing pathways, mitochondrial function, and cellular energy balance, all of which are tightly interconnected with mTOR activity. Therefore, PFNA/PFSA may accelerate DNAm-based aging by enhancing mTOR-driven transcriptional activity, reducing autophagic clearance, and amplifying oxidative and inflammatory stress that subsequently shape methylation patterns embedded in GrimAge, LinAge, and related clocks. Integrating these insights provides a deeper molecular framework supporting the plausibility of PFAS-associated epigenetic aging.

Recent studies have demonstrated that DNAm-based epigenetic clocks, such as GrimAge, offer more precise measures of biological age compared to traditional telomere length assessments (Clement et al., 2022). GrimAge and similar DNAm aging algorithms have shown superior predictive power for mortality and health outcomes, indicating that they capture aging mechanisms more effectively (Tamman et al., 2023; Katrinli et al., 2023). Our findings align with this trend, suggesting that PFAS exposure influences biological aging through pathways that are more readily detected by DNAm biomarkers than by telomere length. This underscores the necessity of integrating DNAm aging algorithms into environmental health studies to elucidate the impact of environmental exposures on aging processes.

In the broader context of environmental health research, our study builds on existing work that has examined the effects of environmental stressors on epigenetic aging. Our study extends this line of inquiry by specifically examining PFAS exposure and identifying sex- and age-specific vulnerabilities. Notably, our findings are consistent with recent results from Khodasevich et al. (2025), who reported sex-specific associations between PFAS exposure and epigenetic age in the NHANES 1999–2000 cohort. In our study, PFNA exposure was particularly associated with accelerated GrimAge-related measures in males, whereas no significant associations were observed in females. Specifically, in the overall population, PFNA was associated with accelerated GrimAgeMortacc (FDR = 0.0209), but not with GrimAge2Mortacc (FDR = 0.0601) or DunedinPoAm (FDR = 0.0629) after FDR adjustment. Age- and sex-stratified analyses showed weaker but directionally consistent associations, with the strongest signals in males (FDR = 0.0742 for GrimAgeMortacc). These results indicate that the associations are generally robust, although some nominally significant findings are attenuated after FDR adjustment. By contrast, Khodasevich et al. reported associations of PFNA with HorvathAge, SkinBloodAge, and PhenoAge. These patterns both corroborate and extend previous work, highlighting that sex-specific epigenetic responses are an important consideration when evaluating PFAS toxicity. Our results further underscore the need for targeted interventions and regulatory actions to mitigate the health impacts of PFAS exposure, particularly in high-risk populations.

While our study provides novel insights into the associations between PFAS (PFNA, PFSA) and epigenetic aging biomarkers, several limitations should be acknowledged. First, the sample size (n = 326) limits the statistical power to detect smaller effect sizes, particularly within stratified subgroups such as sex and age categories. Second, the study population included only adults aged 50 years and older, which restricts the generalizability of our findings to younger age groups. Third, PFAS exposure was assessed during 1999–2000, reflecting a specific historical exposure scenario; the observed associations may not directly represent current exposure profiles or effects in populations with different environmental PFAS patterns. Fourth, the cross-sectional design inherently limits causal inference. While robust associations were observed between PFAS exposure and accelerated epigenetic aging, we cannot establish temporal relationships or rule out reverse causation. For instance, altered biological aging processes may influence PFAS metabolism or excretion rates rather than vice versa. Fifth, FDR adjustment attenuated some nominally significant associations, suggesting potential false positives and indicating that our findings should be interpreted as hypothesis-generating. Future studies with larger, more diverse cohorts, expanded age ranges, updated exposure assessments, and rigorous multiple testing correction are needed for validation.

## 5 Conclusion and implications

Our study identified associations between PFAS exposure, particularly PFNA and PFSA, and accelerated epigenetic aging in older U.S. adults, with patterns differing by sex and age. PFNA was consistently linked to mortality-related aging markers in males and middle-aged individuals, whereas PFSA showed associations with lipid metabolism-related aging measures. These findings persisted after adjustment for sociodemographic, lifestyle, and inflammatory factors, suggesting potential biological relevance. From a public health perspective, our results highlight the importance of considering sex- and age-specific susceptibilities in PFAS monitoring and risk assessment. DNAm aging algorithms, such as GrimAge and LinAge, may serve as valuable biomarkers to detect subclinical impacts of environmental exposures and guide targeted interventions. Future research should focus on longitudinal studies to clarify causal pathways and explore other environmental stressors on biological aging.

## Data Availability

The original contributions presented in the study are included in the article/Supplementary Material, further inquiries can be directed to the corresponding author.
