# Supplementary material for: Emerging PFAS contaminants PFNA and PFSA amplify epigenetic aging: sex- and age-stratified risks in an aging population
Source: Front Aging. 2026 Feb 26;6:1722675. doi: 10.3389/fragi.2025.1722675 (PMC12979462; doi:10.3389/fragi.2025.1722675)
Supplement: Supplementary file 1 [file Table1.docx]

**Supporting Information**

**Emerging PFAS Contaminants PFNA and PFSA Amplify Epigenetic Aging: Sex- and Age-Stratified Risks in an Aging Population**

Ya-Qian Xu ^1, 2^, Chongyu Ding ^1, 2^, Hui Zhang ^1, 2^, Yulu Gong ^1, 2, 3^, Darong Hao ^1, 3^,

Xuetong Zhao ^1, 3^, Kai Li ^4^, Xiangwei Li ^1, 2*^

1. School of Global Health, Chinese Centre for Tropical Diseases Research, Shanghai Jiao Tong University School of Medicine, Shanghai 200025, China.
2. Hainan International Medical Center, Shanghai Jiao Tong University School of Medicine, Hainan 571434, China.
3. School of Public Health, Shanghai Jiao Tong University School of Medicine, Shanghai 200025, China.
4. Department of Ecological Environment, Yangtze Delta Region Institute of Tsinghua University, Zhejiang 314000, China.

**Running title**: PFNA/PFSA Amplify Epigenetic Aging: Sex/Age Risks

**^*^ Correspondence:** Xiangwei Li, e-mail: li.xiangwei@sjtu.edu.cn, Tel: +86 21-63846590-776408

**Word count:** 160 in the abstract; 4000 in the main text.

Number of pages: 12

Number of tables: 6

Number of figure: 1

**Table of Contents**

**Table Captions**

Table S1. Distribution of serum concentrations of perfluoroalkyl substances (ng/mL)

Table S2. Distribution of polyfluoroalkyl chemical in males and females

Table S3. Distribution of polyfluoroalkyl chemical in aged 50-64 and ≥ 65

Table S4. Associations of five polyfluoroalkyl chemicals with twelve DNA methylation algorithms of aging

Table S5. Associations of polyfluoroalkyl chemicals with twelve DNA methylation algorithms of aging by age

Table S6. Associations polyfluoroalkyl chemicals with twelve DNA methylation algorithms of aging by sex

**Figure Caption**

Figure S1. Spearman correlation coefficients of age, polyfluoroalkyl chemicals and twelve DNAm aging algorithms at NHANES 1999-2000 in all participates.

**Table S1. Distribution of** **serum concentrations of** **perfluoroalkyl substances (ng/mL)**

| **Polyfluoroalkyl chemical** | **Synonym** | **LLOD** | **Detection frequency (%)** | **Geometric mean** | **Percentiles** | | | |
| --- | --- | --- | --- | --- | --- | --- | --- | --- |
|  |  |  |  |  | **25th** | **50th** | **75th** | **95th** |
| 2-(N-Ethyl-perfluorooctane sulfonamido) acetic acid | EPAH | 0.2 | 84.66 | 0.53±2.27 | 0.30 | 0.50 | 0.90 | 2.40 |
| 2-(N-Methyl-perfluorooctane sulfonamido) acetic acid | MPAH | 0.17 | 94.17 | 0.62±2.16 | 0.30 | 0.60 | 1.00 | 2.30 |
| Perfluorohexane sulfonic acid | PFHS | 0.1 | 99.69 | 1.84±2.16 | 1.10 | 1.80 | 2.90 | 7.00 |
| Perfluorononanoic acid | PFNA | 0.1 | 95.40 | 0.55±2.20 | 0.40 | 0.50 | 0.90 | 2.00 |
| Perfluorooctanoic acid | PFOA | 0.1 | 100 | 4.08±1.90 | 3.10 | 4.30 | 5.90 | 10.00 |
| Perfluorooctane sulfonic acid | PFOS | 0.2 | 100 | 28.72±1.90 | 20.00 | 29.55 | 43.50 | 75.90 |
| Perfluorooctane sulfonamide | PFSA | 0.05 | 95.71 | 0.29±2.32 | 0.10 | 0.30 | 0.50 | 1.10 |

LLOD: The lower limits of detection;

The detection frequencies of perfluoroundecanoic acid (PFUA), perfluorodecanoic acid (PFDE), perfluorododecanoic acid (PFDO), perfluoroheptanoic acid (PFHP) were 22.09%, 35.89%, 1.23%, and 6.44%, respectively.

**Table S2. Distribution of polyfluoroalkyl chemical in males and females**

| **Polyfluoroalkyl chemical** | **Synonym** | **Males** | | | | | **Females** | | | | | ***P*-value** |
| --- | --- | --- | --- | --- | --- | --- | --- | --- | --- | --- | --- | --- |
|  |  | **Geometric mean** | **25th** | **50th** | **75th** | **95th** | **Geometric mean** | **25th** | **50th** | **75th** | **95th** |  |
| 2-(N-Ethyl-perfluorooctane sulfonamido) acetic acid | EPAH | 0.51±2.20 | 0.30 | 0.50 | 0.90 | 1.90 | 0.55±2.35 | 0.30 | 0.50 | 0.90 | 2.70 | 0.3519 |
| 2-(N-Methyl-perfluorooctane sulfonamido) acetic acid | MPAH | 0.64±2.11 | 0.40 | 0.60 | 1.00 | 2.30 | 0.61±2.22 | 0.30 | 0.50 | 1.00 | 2.00 | 0.9406 |
| Perfluorohexane sulfonic acid | PFHS | 1.89±2.21 | 1.20 | 1.90 | 3.00 | 6.70 | 1.78±2.11 | 1.10 | 1.70 | 2.80 | 7.00 | 0.4850 |
| Perfluorononanoic acid | PFNA | 0.56±2.16 | 0.40 | 0.55 | 1.00 | 1.90 | 0.54±2.25 | 0.30 | 0.50 | 0.90 | 2.00 | 0.9034 |
| Perfluorooctanoic acid | PFOA | 4.07±1.84 | 3.10 | 4.30 | 5.90 | 10.00 | 4.08±1.96 | 3.10 | 4.30 | 5.90 | 9.40 | 0.4220 |
| Perfluorooctane sulfonic acid | PFOS | 29.53±1.90 | 20.65 | 30.20 | 43.00 | 78.60 | 27.89±1.90 | 19.40 | 28.25 | 43.70 | 68.70 | 0.5350 |
| Perfluorooctane sulfonamide | PFSA | 0.28±2.26 | 0.20 | 0.30 | 0.50 | 1.10 | 0.30±2.39 | 0.10 | 0.30 | 0.50 | 1.50 | 0.7764 |

**Table S3. Distribution of polyfluoroalkyl chemical in aged 50-64 and ≥ 65**

| **Polyfluoroalkyl chemical** | **Synonym** | **50-64 years** | | | | | **≥ 65 years** | | | | | ***P*-value** |
| --- | --- | --- | --- | --- | --- | --- | --- | --- | --- | --- | --- | --- |
|  |  | **Geometric mean** | **25th** | **50th** | **75th** | **95th** | **Geometric mean** | **25th** | **50th** | **75th** | **95th** |  |
| 2-(N-Ethyl-perfluorooctane sulfonamido) acetic acid | EPAH | 0.53±2.22 | 0.30 | 0.50 | 0.90 | 2.20 | 0.53±2.32 | 0.30 | 0.40 | 0.90 | 2.80 | 0.5923 |
| 2-(N-Methyl-perfluorooctane sulfonamido) acetic acid | MPAH | 0.63±2.10 | 0.30 | 0.60 | 1.00 | 2.20 | 0.62±2.21 | 0.40 | 0.60 | 1.00 | 2.30 | 0.5505 |
| Perfluorohexane sulfonic acid | PFHS | 1.80±2.17 | 1.10 | 1.80 | 3.00 | 5.80 | 1.87±2.16 | 1.20 | 1.80 | 2.90 | 9.50 | 0.6256 |
| Perfluorononanoic acid | PFNA | 0.53±2.22 | 0.30 | 0.50 | 1.00 | 1.90 | 0.56±2.20 | 0.40 | 0.60 | 0.90 | 2.00 | 0.4632 |
| Perfluorooctanoic acid | PFOA | 4.40±1.95 | 3.30 | 4.40 | 6.10 | 13.00 | 3.85±1.85 | 3.00 | 4.20 | 5.60 | 8.60 | 0.0716 |
| Perfluorooctane sulfonic acid | PFOS | 27.56±1.98 | 19.80 | 28.10 | 42.00 | 69.30 | 29.62±1.84 | 20.30 | 30.30 | 43.50 | 78.50 | 0.4942 |
| Perfluorooctane sulfonamide | PFSA | 0.29±2.35 | 0.20 | 0.30 | 0.50 | 1.10 | 0.29±2.30 | 0.10 | 0.30 | 0.50 | 1.10 | 0.5384 |

**Table S4. Associations of five polyfluoroalkyl chemicals with twelve DNA methylation algorithms of aging**

| **Polyfluoroalkyl chemical** | **Model 1** | | **Model 2** | |
| --- | --- | --- | --- | --- |
|  | **β (95%CI)** | ***P*-value** | **β (95%CI)** | ***P*-value** |
| **EPAH** |  |  |  |  |
| HorvathAgeacc | 0.67 (-1.11,2.44) | 0.4624 | 0.42 (-1.38,2.23) | 0.6464 |
| HannumAgeacc | 1.13 (-0.67,2.92) | 0.2208 | 0.74 (-1.08,2.56) | 0.4250 |
| SkinBloodAgeacc | 0.58 (-1.28,2.44) | 0.5394 | 0.25 (-1.59,2.08) | 0.7920 |
| PhenoAgeacc | 0.11 (-2.00,2.23) | 0.9180 | -0.25 (-2.42,1.93) | 0.8234 |
| LinAgeacc | 2.38 (-0.03,4.80) | 0.0534 | 1.93 (-0.56,4.42) | 0.1290 |
| WeidnerAgeacc | 0.82 (-1.85,3.49) | 0.5454 | 0.63 (-2.16,3.43) | 0.6582 |
| VidalBraloAgeacc | 0.14 (-1.43,1.71) | 0.8604 | 0.00 (-1.68,1.67) | 0.9967 |
| ZhangAgeacc | 0.37 (-0.33,1.06) | 0.3021 | 0.20 (-0.47,0.87) | 0.5569 |
| DunedinPoAm | -0.01 (-0.04,0.01) | 0.3200 | -0.01 (-0.03,0.02) | 0.7134 |
| HorvathTelo | -0.05 (-0.12,0.03) | 0.2216 | -0.05 (-0.13,0.03) | 0.2306 |
| GrimAgeMortacc | -0.56 (-1.86,0.73) | 0.3948 | -0.50 (-1.90,0.90) | 0.4851 |
| GrimAge2Mortacc | -0.93 (-2.36,0.50) | 0.2024 | -0.86 (-2.38,0.65) | 0.2655 |
| **MPAH** |  |  |  |  |
| HorvathAgeacc | 0.89 (-1.01,2.79) | 0.3585 | 0.68 (-1.27,2.64) | 0.4931 |
| HannumAgeacc | 0.97 (-0.96,2.90) | 0.3252 | 0.60 (-1.37,2.57) | 0.5526 |
| SkinBloodAgeacc | 0.62 (-1.37,2.61) | 0.5388 | 0.28 (-1.71,2.27) | 0.7817 |
| PhenoAgeacc | -0.65 (-2.91,1.62) | 0.5749 | -1.17 (-3.52,1.18) | 0.3309 |
| LinAgeacc | 0.04 (-2.56,2.63) | 0.9783 | 0.01 (-2.70,2.72) | 0.9957 |
| WeidnerAgeacc | 1.91 (-0.94,4.76) | 0.1907 | 0.97 (-2.06,4.00) | 0.5310 |
| VidalBraloAgeacc | 1.00 (-0.69,2.68) | 0.2467 | 0.47 (-1.34,2.29) | 0.6115 |
| ZhangAgeacc | 0.04 (-0.70,0.79) | 0.9067 | -0.06 (-0.78,0.66) | 0.8644 |
| DunedinPoAm | -0.01 (-0.04,0.02) | 0.5487 | 0.00 (-0.03,0.03) | 0.9302 |
| HorvathTelo | -0.02 (-0.10,0.06) | 0.5759 | 0.01 (-0.07,0.10) | 0.7902 |
| GrimAgeMortacc | 0.22 (-1.17,1.61) | 0.7550 | -0.02 (-1.54,1.49) | 0.9753 |
| GrimAge2Mortacc | -0.06 (-1.59,1.47) | 0.9351 | -0.38 (-2.02,1.27) | 0.6541 |
| **PFHS** |  |  |  |  |
| HorvathAgeacc | 0.76 (-0.55,2.07) | 0.2570 | 0.71 (-0.63,2.06) | 0.2984 |
| HannumAgeacc | 0.17 (-1.16,1.50) | 0.8006 | 0.35 (-1.00,1.71) | 0.6095 |
| SkinBloodAgeacc | 0.57 (-0.80,1.95) | 0.4125 | 0.42 (-0.95,1.79) | 0.5454 |
| PhenoAgeacc | 0.48 (-1.08,2.04) | 0.5442 | 0.28 (-1.35,1.90) | 0.7384 |
| LinAgeacc | 1.00 (-0.79,2.78) | 0.2752 | 0.79 (-1.07,2.65) | 0.4069 |
| WeidnerAgeacc | 1.32 (-0.65,3.28) | 0.1907 | 1.06 (-1.03,3.14) | 0.3213 |
| VidalBraloAgeacc | 0.54 (-0.62,1.70) | 0.3605 | 0.26 (-0.99,1.52) | 0.6788 |
| ZhangAgeacc | 0.19 (-0.32,0.71) | 0.4607 | 0.11 (-0.39,0.61) | 0.6678 |
| DunedinPoAm | 0.00 (-0.02,0.02) | 0.9253 | 0.00 (-0.02,0.02) | 0.7049 |
| HorvathTelo | -0.02 (-0.08,0.03) | 0.4574 | -0.03 (-0.09,0.03) | 0.3065 |
| GrimAgeMortacc | 0.32 (-0.64,1.27) | 0.5167 | 0.25 (-0.80,1.29) | 0.6422 |
| GrimAge2Mortacc | -0.22 (-1.27,0.84) | 0.6866 | -0.24 (-1.37,0.90) | 0.6846 |

**Table S4. Associations of five polyfluoroalkyl chemicals with twelve DNA methylation algorithms of aging (continued)**

| **Polyfluoroalkyl chemical** | **Model 1** | | **Model 2** | |
| --- | --- | --- | --- | --- |
|  | **β (95%CI)** | ***P*-value** | **β (95%CI)** | ***P*-value** |
| **PFOA** |  |  |  |  |
| HorvathAgeacc | 0.67 (-0.69,2.04) | 0.3332 | 0.35 (-1.06,1.76) | 0.6264 |
| HannumAgeacc | 0.62 (-0.77,2.00) | 0.3819 | 0.63 (-0.80,2.05) | 0.3897 |
| SkinBloodAgeacc | 0.97 (-0.46,2.39) | 0.1839 | 0.68 (-0.75,2.12) | 0.3522 |
| PhenoAgeacc | -0.06 (-1.69,1.56) | 0.9377 | -0.24 (-1.94,1.47) | 0.7853 |
| LinAgeacc | 0.89 (-0.97,2.75) | 0.3495 | 0.59 (-1.37,2.55) | 0.5549 |
| WeidnerAgeacc | 1.60 (-0.45,3.64) | 0.1270 | 1.00 (-1.19,3.18) | 0.3717 |
| VidalBraloAgeacc | 0.80 (-0.41,2.00) | 0.1968 | 0.25 (-1.06,1.56) | 0.7103 |
| ZhangAgeacc | 0.22 (-0.31,0.76) | 0.4179 | 0.07 (-0.45,0.59) | 0.7942 |
| DunedinPoAm | 0.00 (-0.02,0.02) | 0.7291 | 0.01 (-0.01,0.03) | 0.2737 |
| HorvathTelo | -0.02 (-0.08,0.03) | 0.4104 | -0.04 (-0.10,0.03) | 0.2440 |
| GrimAgeMortacc | 0.55 (-0.45,1.54) | 0.2831 | 0.64 (-0.45,1.74) | 0.2500 |
| GrimAge2Mortacc | 0.02 (-1.08,1.11) | 0.9780 | 0.24 (-0.95,1.43) | 0.6889 |
| **PFOS** |  |  |  |  |
| HorvathAgeacc | 0.59 (-0.49,1.68) | 0.2836 | 0.33 (-0.84,1.50) | 0.5836 |
| HannumAgeacc | 0.45 (-0.65,1.55) | 0.4253 | 0.50 (-0.68,1.68) | 0.4072 |
| SkinBloodAgeacc | 0.99 (-0.15,2.12) | 0.0885 | 0.84 (-0.35,2.03) | 0.1694 |
| PhenoAgeacc | 0.58 (-0.72,1.87) | 0.3818 | 0.40 (-1.01,1.82) | 0.5763 |
| LinAgeacc | 1.18 (-0.29,2.66) | 0.1169 | 1.30 (-0.31,2.92) | 0.1147 |
| WeidnerAgeacc | 0.68 (-0.95,2.32) | 0.4117 | 0.37 (-1.45,2.19) | 0.6905 |
| VidalBraloAgeacc | 0.37 (-0.59,1.33) | 0.4549 | 0.02 (-1.07,1.11) | 0.9747 |
| ZhangAgeacc | 0.29 (-0.13,0.72) | 0.1800 | 0.23 (-0.20,0.66) | 0.2997 |
| DunedinPoAm | 0.00 (-0.01,0.02) | 0.6326 | 0.01 (-0.01,0.03) | 0.2152 |
| HorvathTelo | 0.00 (-0.04,0.05) | 0.9486 | -0.01 (-0.07,0.04) | 0.6040 |
| GrimAgeMortacc | 0.44 (-0.36,1.23) | 0.2817 | 0.55 (-0.36,1.46) | 0.2355 |
| GrimAge2Mortacc | 0.11 (-0.76,0.99) | 0.8019 | 0.31 (-0.68,1.30) | 0.5377 |

Model 1: adjusted for age, sex, and white blood cell count;

Model 2: adjusted for age, sex, white blood cell count, race, educational status, poverty income ratio, body mass index, and C-reactive protein

**Table S5. Associations of polyfluoroalkyl chemicals with twelve DNA methylation algorithms of aging by age**

| **Polyfluoroalkyl chemical** | **50-64 years** | | **≥ 65 years** | | ***P*-^interaction^** |
| --- | --- | --- | --- | --- | --- |
|  | **β (95%CI)** | ***P*-value** | **β (95%CI)** | ***P*-value** |  |
| **EPAH** |  |  |  |  |  |
| HorvathAgeacc | -0.62 (-3.55,2.31) | 0.6797 | 0.95 (-1.45,3.35) | 0.4389 | 0.5771 |
| HannumAgeacc | -1.28 (-4.18,1.63) | 0.3909 | 1.62 (-0.77,4.00) | 0.1858 | 0.2534 |
| SkinBloodAgeacc | -1.53 (-3.97,0.91) | 0.2210 | 1.23 (-1.36,3.82) | 0.3540 | 0.5837 |
| PhenoAgeacc | -2.44 (-5.68,0.81) | 0.1447 | 0.64 (-2.30,3.59) | 0.6691 | 0.8413 |
| LinAgeacc | -0.98 (-4.94,2.98) | 0.6293 | 3.41 (0.15,6.67) | 0.0424 | 0.0853 |
| WeidnerAgeacc | -1.59 (-5.67,2.49) | 0.4470 | 2.40 (-1.41,6.20) | 0.2198 | 0.5720 |
| VidalBraloAgeacc | -1.28 (-3.77,1.20) | 0.3128 | 0.85 (-1.43,3.13) | 0.4671 | 0.9798 |
| ZhangAgeacc | -0.41 (-1.25,0.43) | 0.3422 | 0.49 (-0.46,1.44) | 0.3115 | 0.3875 |
| DunedinPoAm | -0.02 (-0.07,0.03) | 0.4498 | -0.01 (-0.04,0.02) | 0.6888 | 0.7965 |
| HorvathTelo | 0.00 (-0.13,0.14) | 0.9859 | -0.07 (-0.17,0.02) | 0.1417 | 0.1635 |
| GrimAgeMortacc | -1.34 (-4.02,1.34) | 0.3308 | -0.34 (-1.97,1.30) | 0.6872 | 0.7507 |
| GrimAge2Mortacc | -1.71 (-4.52,1.10) | 0.2362 | -0.74 (-2.52,1.05) | 0.4216 | 0.4815 |
| **MPAH** |  |  |  |  |  |
| HorvathAgeacc | 2.95 (-0.19,6.10) | 0.0687 | -0.45 (-3.14,2.23) | 0.7411 | 0.9308 |
| HannumAgeacc | 2.19 (-0.96,5.34) | 0.1752 | -0.06 (-2.74,2.62) | 0.9649 | 0.7460 |
| SkinBloodAgeacc | 1.96 (-0.69,4.62) | 0.1497 | -0.13 (-3.04,2.77) | 0.9283 | 0.9995 |
| PhenoAgeacc | -1.25 (-4.82,2.32) | 0.4954 | -1.56 (-4.85,1.72) | 0.3526 | 0.3348 |
| LinAgeacc | 1.51 (-2.80,5.82) | 0.4945 | -0.71 (-4.41,2.99) | 0.7072 | 0.6964 |
| WeidnerAgeacc | 3.15 (-1.27,7.57) | 0.1651 | 1.07 (-3.21,5.35) | 0.6246 | 0.8193 |
| VidalBraloAgeacc | 2.80 (0.14,5.47) | 0.0418 | -0.09 (-2.65,2.46) | 0.9441 | 0.8529 |
| ZhangAgeacc | 0.61 (-0.30,1.52) | 0.1931 | -0.36 (-1.42,0.70) | 0.5054 | 0.6488 |
| DunedinPoAm | 0.03 (-0.03,0.08) | 0.3462 | -0.02 (-0.06,0.01) | 0.1850 | 0.7074 |
| HorvathTelo | -0.07 (-0.22,0.08) | 0.3552 | 0.04 (-0.07,0.15) | 0.4520 | 0.6438 |
| GrimAgeMortacc | 2.00 (-0.91,4.91) | 0.1805 | -0.76 (-2.58,1.07) | 0.4167 | 0.8281 |
| GrimAge2Mortacc | 1.38 (-1.69,4.46) | 0.3800 | -1.10 (-3.10,0.90) | 0.2817 | 0.5680 |
| **PFHS** |  |  |  |  |  |
| HorvathAgeacc | -0.49 (-2.57,1.59) | 0.6461 | 1.16 (-0.82,3.15) | 0.2532 | 0.3082 |
| HannumAgeacc | -1.06 (-3.12,1.01) | 0.3182 | 1.05 (-0.93,3.04) | 0.2992 | 0.3750 |
| SkinBloodAgeacc | -1.61 (-3.34,0.11) | 0.0685 | 1.44 (-0.70,3.58) | 0.1878 | 0.3242 |
| PhenoAgeacc | -1.25 (-3.58,1.07) | 0.2918 | 0.45 (-1.99,2.89) | 0.7183 | 0.5786 |
| LinAgeacc | -1.72 (-4.52,1.08) | 0.2316 | 1.61 (-1.12,4.34) | 0.2491 | 0.3454 |
| WeidnerAgeacc | -1.35 (-4.25,1.55) | 0.3640 | 3.07 (-0.06,6.21) | 0.0565 | 0.3181 |
| VidalBraloAgeacc | -1.33 (-3.09,0.42) | 0.1392 | 1.04 (-0.85,2.92) | 0.2836 | 0.6924 |
| ZhangAgeacc | -0.45 (-1.04,0.15) | 0.1417 | 0.32 (-0.47,1.10) | 0.4313 | 0.4384 |
| DunedinPoAm | 0.03 (-0.01,0.06) | 0.1476 | -0.01 (-0.03,0.02) | 0.5608 | 0.9960 |
| HorvathTelo | -0.04 (-0.14,0.05) | 0.3788 | -0.02 (-0.11,0.06) | 0.5830 | 0.3162 |
| GrimAgeMortacc | 0.65 (-1.26,2.56) | 0.5059 | 0.29 (-1.06,1.64) | 0.6746 | 0.5679 |
| GrimAge2Mortacc | 0.28 (-1.73,2.30) | 0.7822 | -0.43 (-1.91,1.06) | 0.5724 | 0.8174 |

**Table S5. Associations of polyfluoroalkyl chemicals with twelve DNA methylation algorithms of aging by age (continued)**

| **Polyfluoroalkyl chemical** | **50-64 years** | | **≥ 65 years** | | ***P*-^interaction^** | |
| --- | --- | --- | --- | --- | --- | --- |
|  | **β (95%CI)** | ***P*-value** | **β (95%CI)** | ***P*-value** | |  |
| **PFOA** |  |  |  |  | |  |
| HorvathAgeacc | 0.08 (-1.79,1.95) | 0.9347 | 0.55 (-1.65,2.75) | 0.6243 | | 0.7742 |
| HannumAgeacc | 0.18 (-1.68,2.04) | 0.8483 | 0.87 (-1.32,3.06) | 0.4359 | | 0.3939 |
| SkinBloodAgeacc | -0.35 (-1.91,1.22) | 0.6659 | 1.68 (-0.68,4.04) | 0.1640 | | 0.3152 |
| PhenoAgeacc | -0.79 (-2.88,1.30) | 0.4586 | -0.20 (-2.89,2.50) | 0.8872 | | 0.9759 |
| LinAgeacc | -0.51 (-3.04,2.02) | 0.6944 | 1.49 (-1.53,4.50) | 0.3346 | | 0.5870 |
| WeidnerAgeacc | -0.21 (-2.82,2.40) | 0.8760 | 2.94 (-0.53,6.41) | 0.0986 | | 0.5726 |
| VidalBraloAgeacc | -1.03 (-2.61,0.55) | 0.2048 | 1.60 (-0.47,3.67) | 0.1326 | | 0.7903 |
| ZhangAgeacc | -0.16 (-0.70,0.38) | 0.5678 | 0.21 (-0.65,1.08) | 0.6297 | | 0.6900 |
| DunedinPoAm | 0.02 (-0.02,0.05) | 0.2873 | -0.01 (-0.04,0.02) | 0.4656 | | 0.6433 |
| HorvathTelo | -0.03 (-0.12,0.05) | 0.4482 | -0.04 (-0.13,0.05) | 0.3614 | | 0.2724 |
| GrimAgeMortacc | 0.83 (-0.89,2.54) | 0.3464 | 0.15 (-1.34,1.64) | 0.8445 | | 0.2959 |
| GrimAge2Mortacc | 0.69 (-1.12,2.49) | 0.4564 | -0.68 (-2.32,0.95) | 0.4129 | | 0.7424 |
| **PFOS** |  |  |  |  | |  |
| HorvathAgeacc | -0.50 (-2.24,1.23) | 0.5713 | 0.67 (-1.03,2.36) | 0.4428 | | 0.8815 |
| HannumAgeacc | -0.56 (-2.28,1.16) | 0.5265 | 0.90 (-0.79,2.59) | 0.2983 | | 0.4778 |
| SkinBloodAgeacc | -0.46 (-1.91,0.99) | 0.5363 | 1.52 (-0.30,3.34) | 0.1035 | | 0.3352 |
| PhenoAgeacc | -0.76 (-2.70,1.18) | 0.4448 | 0.86 (-1.22,2.94) | 0.4175 | | 0.5694 |
| LinAgeacc | -0.73 (-3.07,1.62) | 0.5451 | 2.17 (-0.14,4.48) | 0.0674 | | 0.3495 |
| WeidnerAgeacc | -1.05 (-3.46,1.37) | 0.3968 | 1.62 (-1.07,4.31) | 0.2404 | | 0.8303 |
| VidalBraloAgeacc | -1.38 (-2.83,0.07) | 0.0656 | 0.81 (-0.80,2.42) | 0.3247 | | 0.6657 |
| ZhangAgeacc | -0.18 (-0.68,0.32) | 0.4866 | 0.36 (-0.31,1.03) | 0.2967 | | 0.4271 |
| DunedinPoAm | 0.02 (-0.01,0.05) | 0.1306 | 0.00 (-0.02,0.02) | 0.9542 | | 0.5413 |
| HorvathTelo | -0.01 (-0.09,0.07) | 0.8173 | -0.01 (-0.08,0.06) | 0.8149 | | 0.6861 |
| GrimAgeMortacc | 1.01 (-0.58,2.59) | 0.2157 | 0.42 (-0.73,1.57) | 0.4767 | | 0.2689 |
| GrimAge2Mortacc | 0.79 (-0.88,2.46) | 0.3568 | 0.08 (-1.18,1.35) | 0.8957 | | 0.4757 |

Liner regressions were adjusted for sex, white blood cell count, race, educational status, poverty income ratio, and body mass index, C-reactive protein.

**Table S6. Associations polyfluoroalkyl chemicals with twelve DNA methylation algorithms of aging by sex**

| **Polyfluoroalkyl chemical** | **Male** | | **Female** | | ***P*-^interaction^** |
| --- | --- | --- | --- | --- | --- |
|  | **β (95%CI)** | ***P*-value** | **β (95%CI)** | ***P*-value** |  |
| **EPAH** |  |  |  |  |  |
| HorvathAgeacc | 1.00 (-2.06,4.06) | 0.5214 | -1.06 (-3.10,0.99) | 0.3142 | 0.9060 |
| HannumAgeacc | 0.11 (-3.01,3.22) | 0.9465 | 0.61 (-1.55,2.76) | 0.5833 | 0.3904 |
| SkinBloodAgeacc | 0.92 (-2.46,4.30) | 0.5930 | -0.87 (-2.80,1.06) | 0.3802 | 0.9731 |
| PhenoAgeacc | -1.14 (-4.68,2.39) | 0.5273 | -0.51 (-3.18,2.16) | 0.7074 | 0.8788 |
| LinAgeacc | -0.07 (-4.30,4.16) | 0.9738 | 2.60 (-0.28,5.49) | 0.0796 | 0.0795 |
| WeidnerAgeacc | 0.01 (-4.45,4.48) | 0.9960 | 0.54 (-2.98,4.06) | 0.7652 | 0.5958 |
| VidalBraloAgeacc | 1.01 (-1.84,3.87) | 0.4888 | -1.09 (-3.06,0.89) | 0.2840 | 0.7653 |
| ZhangAgeacc | 0.35 (-0.90,1.59) | 0.5891 | -0.05 (-0.70,0.61) | 0.8910 | 0.6941 |
| DunedinPoAm | 0.00 (-0.04,0.04) | 0.9987 | -0.01 (-0.05,0.02) | 0.5300 | 0.6985 |
| HorvathTelo | -0.03 (-0.16,0.10) | 0.6681 | -0.06 (-0.16,0.03) | 0.1965 | 0.2074 |
| GrimAgeMortacc | 0.27 (-2.08,2.63) | 0.8204 | -0.97 (-2.69,0.75) | 0.2700 | 0.3944 |
| GrimAge2Mortacc | -0.11 (-2.56,2.34) | 0.9299 | -1.44 (-3.38,0.49) | 0.1465 | 0.2146 |
| **MPAH** |  |  |  |  |  |
| HorvathAgeacc | 1.87 (-1.50,5.23) | 0.2795 | -0.27 (-2.53,1.99) | 0.8152 | 0.8494 |
| HannumAgeacc | 0.84 (-2.60,4.27) | 0.6344 | 0.15 (-2.22,2.53) | 0.8997 | 0.7394 |
| SkinBloodAgeacc | 0.47 (-3.26,4.21) | 0.8049 | -0.45 (-2.58,1.68) | 0.6795 | 0.9743 |
| PhenoAgeacc | -0.87 (-4.78,3.04) | 0.6643 | -1.70 (-4.62,1.22) | 0.2550 | 0.2571 |
| LinAgeacc | -1.08 (-5.75,3.59) | 0.6511 | 0.61 (-2.61,3.82) | 0.7121 | 0.9974 |
| WeidnerAgeacc | -0.54 (-5.47,4.39) | 0.8314 | 1.00 (-2.87,4.86) | 0.6148 | 0.5562 |
| VidalBraloAgeacc | 0.76 (-2.40,3.92) | 0.6379 | -0.18 (-2.36,2.01) | 0.8737 | 0.7991 |
| ZhangAgeacc | 0.05 (-1.34,1.43) | 0.9489 | -0.37 (-1.09,0.35) | 0.3141 | 0.6736 |
| DunedinPoAm | -0.01 (-0.06,0.04) | 0.6367 | 0.01 (-0.03,0.05) | 0.6663 | 0.7301 |
| HorvathTelo | 0.05 (-0.09,0.20) | 0.4858 | 0.00 (-0.11,0.10) | 0.9285 | 0.8689 |
| GrimAgeMortacc | 0.91 (-1.69,3.50) | 0.4939 | -0.70 (-2.59,1.20) | 0.4726 | 0.7605 |
| GrimAge2Mortacc | 0.60 (-2.10,3.31) | 0.6634 | -1.17 (-3.31,0.97) | 0.2854 | 0.4747 |
| **PFHS** |  |  |  |  |  |
| HorvathAgeacc | 1.77 (-0.29,3.84) | 0.0940 | -1.02 (-2.72,0.68) | 0.2420 | 0.7169 |
| HannumAgeacc | 0.94 (-1.17,3.05) | 0.3851 | -0.96 (-2.75,0.83) | 0.2947 | 0.8872 |
| SkinBloodAgeacc | 1.19 (-1.11,3.48) | 0.3120 | -0.58 (-2.19,1.03) | 0.4789 | 0.8427 |
| PhenoAgeacc | 0.95 (-1.45,3.36) | 0.4381 | -1.20 (-3.41,1.01) | 0.2893 | 0.9393 |
| LinAgeacc | 0.80 (-2.07,3.68) | 0.5853 | -0.47 (-2.90,1.96) | 0.7062 | 0.5309 |
| WeidnerAgeacc | 2.71 (-0.30,5.71) | 0.0796 | -1.45 (-4.36,1.47) | 0.3337 | 0.5875 |
| VidalBraloAgeacc | 0.89 (-1.05,2.83) | 0.3705 | -1.01 (-2.65,0.63) | 0.2292 | 0.9110 |
| ZhangAgeacc | 0.34 (-0.51,1.19) | 0.4377 | -0.28 (-0.82,0.27) | 0.3222 | 0.9368 |
| DunedinPoAm | 0.01 (-0.02,0.03) | 0.7299 | 0.01 (-0.02,0.04) | 0.4560 | 0.5855 |
| HorvathTelo | -0.04 (-0.13,0.05) | 0.4125 | -0.01 (-0.09,0.07) | 0.8051 | 0.3733 |
| GrimAgeMortacc | 0.41 (-1.19,2.01) | 0.6134 | 0.08 (-1.35,1.52) | 0.9087 | 0.6384 |
| GrimAge2Mortacc | -0.26 (-1.93,1.41) | 0.7609 | -0.14 (-1.76,1.49) | 0.8689 | 0.8116 |

**Table S6. Associations of polyfluoroalkyl chemicals with twelve DNA methylation algorithms of aging by sex (continued)**

| **Polyfluoroalkyl chemical** | **Male** | | | **Female** | | ***P*-^interaction^** | |
| --- | --- | --- | --- | --- | --- | --- | --- |
|  | **β (95%CI)** | ***P*-value** | **β (95%CI)** | | ***P*-value** | |  |
| **PFOA** |  |  |  | |  | |  |
| HorvathAgeacc | 0.46 (-1.96,2.87) | 0.7128 | -0.40 (-2.04,1.23) | | 0.6292 | | 0.8115 |
| HannumAgeacc | 0.68 (-1.78,3.13) | 0.5889 | -0.03 (-1.75,1.69) | | 0.9736 | | 0.4720 |
| SkinBloodAgeacc | 0.96 (-1.71,3.62) | 0.4824 | 0.03 (-1.51,1.58) | | 0.9661 | | 0.4925 |
| PhenoAgeacc | -1.28 (-4.07,1.51) | 0.3715 | -0.39 (-2.52,1.73) | | 0.7165 | | 0.8200 |
| LinAgeacc | -0.50 (-3.84,2.84) | 0.7702 | 0.36 (-1.96,2.69) | | 0.7600 | | 0.4872 |
| WeidnerAgeacc | 1.52 (-1.99,5.04) | 0.3975 | -0.30 (-3.10,2.50) | | 0.8337 | | 0.3969 |
| VidalBraloAgeacc | 0.77 (-1.49,3.02) | 0.5060 | -0.69 (-2.26,0.89) | | 0.3935 | | 0.8400 |
| ZhangAgeacc | 0.08 (-0.90,1.07) | 0.8666 | -0.14 (-0.66,0.38) | | 0.6063 | | 0.8869 |
| DunedinPoAm | 0.00 (-0.03,0.04) | 0.8275 | 0.02 (0.00,0.05) | | 0.0931 | | 0.1528 |
| HorvathTelo | -0.01 (-0.11,0.10) | 0.8879 | -0.04 (-0.12,0.04) | | 0.2995 | | 0.1857 |
| GrimAgeMortacc | 0.97 (-0.89,2.82) | 0.3089 | 0.48 (-0.89,1.86) | | 0.4902 | | 0.2621 |
| GrimAge2Mortacc | 0.21 (-1.72,2.15) | 0.8291 | 0.34 (-1.21,1.89) | | 0.6670 | | 0.5911 |
| **PFOS** |  |  |  | |  | |  |
| HorvathAgeacc | 0.74 (-1.13,2.62) | 0.4394 | -0.16 (-1.57,1.24) | | 0.8213 | | 0.8406 |
| HannumAgeacc | 0.99 (-0.92,2.89) | 0.3124 | -0.04 (-1.52,1.44) | | 0.9568 | | 0.5670 |
| SkinBloodAgeacc | 1.31 (-0.75,3.38) | 0.2151 | 0.23 (-1.09,1.56) | | 0.7326 | | 0.2995 |
| PhenoAgeacc | 0.08 (-2.10,2.25) | 0.9445 | 0.06 (-1.76,1.89) | | 0.9451 | | 0.5950 |
| LinAgeacc | 0.83 (-1.76,3.42) | 0.5309 | 1.64 (-0.34,3.62) | | 0.1064 | | 0.0997 |
| WeidnerAgeacc | 0.80 (-1.94,3.54) | 0.5657 | 0.38 (-2.03,2.78) | | 0.7608 | | 0.6381 |
| VidalBraloAgeacc | 0.04 (-1.72,1.80) | 0.9631 | -0.09 (-1.45,1.27) | | 0.9001 | | 0.8633 |
| ZhangAgeacc | 0.45 (-0.31,1.21) | 0.2504 | -0.02 (-0.47,0.43) | | 0.9422 | | 0.4806 |
| DunedinPoAm | 0.01 (-0.02,0.04) | 0.4651 | 0.01 (-0.01,0.04) | | 0.2238 | | 0.1750 |
| HorvathTelo | -0.03 (-0.11,0.05) | 0.4835 | 0.00 (-0.07,0.07) | | 0.9591 | | 0.6887 |
| GrimAgeMortacc | 1.06 (-0.37,2.50) | 0.1482 | 0.21 (-0.97,1.39) | | 0.7273 | | 0.3136 |
| GrimAge2Mortacc | 0.72 (-0.78,2.22) | 0.3468 | 0.01 (-1.33,1.35) | | 0.9884 | | 0.5946 |
| **PFSA** |  |  |  | |  | |  |
| HorvathAgeacc | 0.67 (-3.28,4.62) | 0.7398 | -0.12 (-3.50,3.25) | | 0.9440 | | 0.6885 |
| HannumAgeacc | 0.76 (-3.26,4.77) | 0.7129 | -0.13 (-3.68,3.41) | | 0.9411 | | 0.7677 |
| SkinBloodAgeacc | 0.65 (-3.72,5.01) | 0.7722 | -0.18 (-3.36,3.01) | | 0.9133 | | 0.6557 |
| PhenoAgeacc | -0.21 (-4.78,4.37) | 0.9296 | -1.71 (-6.08,2.66) | | 0.4449 | | 0.7527 |
| LinAgeacc | 3.58 (-1.84,9.00) | 0.1976 | 4.26 (-0.48,9.00) | | 0.0804 | | 0.0350 |
| WeidnerAgeacc | 2.61 (-3.14,8.35) | 0.3755 | -0.14 (-5.92,5.64) | | 0.9620 | | 0.6429 |
| VidalBraloAgeacc | 2.12 (-1.55,5.80) | 0.2592 | -0.14 (-3.40,3.12) | | 0.9315 | | 0.4625 |
| ZhangAgeacc | 0.75 (-0.86,2.36) | 0.3628 | -0.18 (-1.26,0.89) | | 0.7375 | | 0.4825 |
| DunedinPoAm | -0.02 (-0.07,0.04) | 0.5177 | 0.00 (-0.06,0.06) | | 0.9970 | | 0.8101 |
| HorvathTelo | 0.04 (-0.14,0.21) | 0.6858 | -0.01 (-0.17,0.15) | | 0.9392 | | 0.8857 |
| GrimAgeMortacc | -1.07 (-4.10,1.97) | 0.4915 | -0.37 (-3.20,2.46) | | 0.7997 | | 0.5811 |
| GrimAge2Mortacc | -1.22 (-4.37,1.94) | 0.4513 | -1.49 (-4.68,1.71) | | 0.3628 | | 0.3272 |

Liner regressions were adjusted for age, white blood cell count, race, educational status, poverty income ratio, and body mass index, C-reactive protein.

| 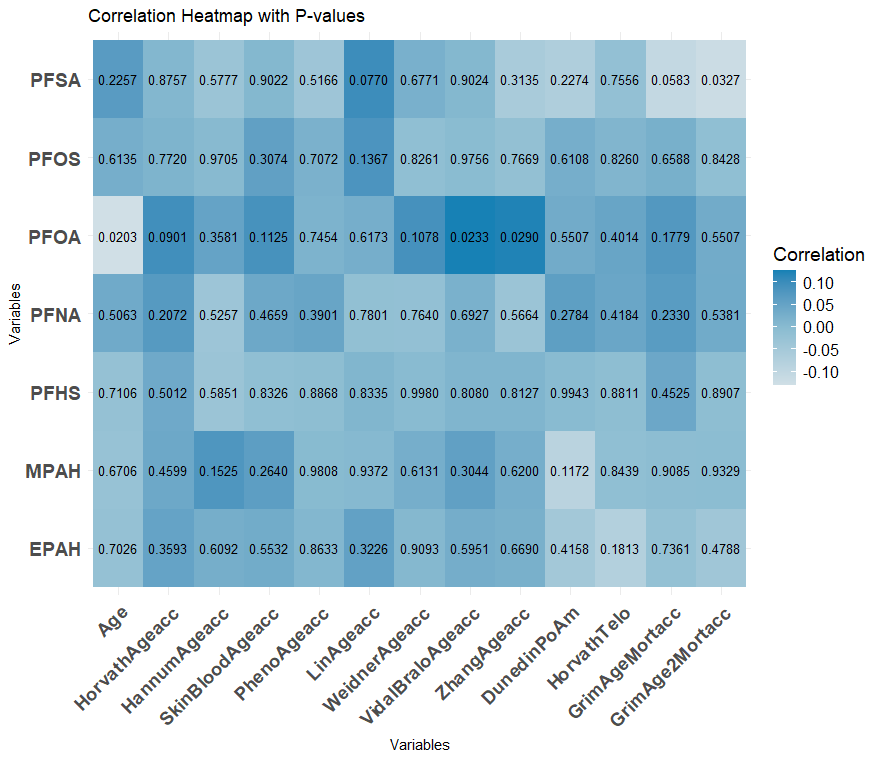 |
| --- |

Figure S1. Spearman correlation coefficients of age, polyfluoroalkyl chemicals and twelve DNAm aging algorithms at NHANES 1999-2000 in all participates.
